# The Diary of a Late Physician

**Published:** 1905-09

**Authors:** 


### Books and Magazines.

OUR READERS will be interested in the forthcoming publication of "A Manual and Atlas of Orthopedic Surgery," by Dr. James K., Young, Professor of Orthopedic Surgery, Philadelphia Polyclinic, etc. We believe that this work is destined to occupy a very important place in medical literature. It is to be a quarto in size, of about 900 pages, and will contain upwards of 800 illustrations. A definite price for the complete work has not yet been fixed, but all advance orders received will be filled by the publishers, as soon as the book is ready, at $10 for cloth binding, and $12 for half morocco binding. P. Blakiston's Son & Co., are the publishers. Mention the JOURNAL.

---

THE DIARY OF A LATE PHYSICIAN.—By Samuel Warren, D. C. L., F. R. S. Arranged by Charles Wells Moulton. Pages 379. Illustrated. Silk cloth. Price, $2.50. The Saalfield Publishing Co., Akron, Ohio.

This is the latest volume in the Doctor's Recreation Series and is an excellent addition to this set of books. The author, Samuel Warren, was born in Denbighshire, England, in 1807, and died in 1877. He studied both medicine and law; was made a Q. C. in 1851; was Recorder of Hull, 1852-1874; was a conservative member of parliament, 1856-59, and then Master of Lunacy. He was the author of a number of books in addition to this work. "The Diary of a Late Physician" was first published in serial form in Blackwood's magazine and attracted much attention. It was published in book form in several languages and many editions were sold. As the work has been out of print for some time the present edition will be welcomed.

Dr. Warren's story starts out with the early struggles of a young physician in London. The hard battle which he fought before he finally obtained a very lucrative practice, will be appreciated by every physician as he recalls the early days of his own practice. The book recites the many exciting and tragic events which the physician of large practice met in his life's work. The stories are told in a powerful and direct style and are strong presentations of many pages from the vast book of human suffering.

Every physician should have the Doctor's Recreation Series in his library for it contains many medico-literary classics to be obtained nowhere else. The volumes are beautifully bound in brown silk with gilt tops. Each volume contains four photo-gravaure reproductions of famous paintings appropriate to the work.—*Exchange.*

Dr. Warren was the author of the best novel in the English language, "Ten Thousand a Year." This famous book has recently been brought out under the name of "Tittlebat Titmouse."

